# The Mediating Effects of Nursing Professional Commitment on the Relationship between Social Support, Resilience, and Intention to Stay among Newly Graduated Male Nurses: A Cross-Sectional Questionnaire Survey

**DOI:** 10.3390/ijerph18147546

**Published:** 2021-07-15

**Authors:** Hsingyi Yu, Chunhsia Huang, Yenfan Chin, Yungchao Shen, Yuehtao Chiang, Chiwen Chang, Jiunnhorng Lou

**Affiliations:** 1 School of Nursing, College of Medicine, Chang-Gung University, Taoyuan 333323, Taiwan; chhuang@mail.cgu.edu.tw (C.H.); yenfan@mail.cgu.edu.tw (Y.C.); lisachiang@mail.cgu.edu.tw (Y.C.); cwchang@mail.cgu.edu.tw (C.C.); 2 Department of Nursing, Chang-Gung Memorial Hospital, Taoyuan 333423, Taiwan; 3 Department of Endocrinology and Metabolism, Linkou Branch, Chang-Gung Memorial Hospital, Taoyuan 333423, Taiwan; 4 Department of Nursing, New Taipei Municipal Tu Cheng Hospital, New Taipei 236017, Taiwan; q12185@cgmh.org.tw; 5 Division of Pediatric Endocrinology & Genetics, Department of Pediatrics, Chang-Gung Memorial Hospital, Taoyuan 333423, Taiwan; 6 Department of Nursing, Hsin Sheng College of Medical Care and Management, Taoyuan 325004, Taiwan

**Keywords:** newly graduated male nurses, intention to stay, nursing professional commitment, Job Demands-Resources (JD-R) model

## Abstract

The current shortage of nurses is an important global issue. Most male nurses leave nursing within four years of starting their nursing career. It is crucial to understand the influencing factors on newly graduated male nurses staying in nursing. Previous studies on intentions to stay as nurses were seldom based on theory and failed to consider the differences between genders. Based on the Job Demands-Resources (JD-R) model, this study tested the model that social support, resilience, and nursing professional commitment influence the intention to stay and the mediating effect of nursing professional commitment in the above relationship. This cross-sectional study adopted purposive and snowball sampling methods. Data were collected using online questionnaire, and 272 newly graduated male nurses completed it. The hypothetical model had a good fit with the data. Nursing professional commitment had a complete mediating effect between social support and intention to stay and between resilience and intention to stay. Nursing professional commitment was highly positively correlated to intention to stay. It is suggested that future research and practice should enhance male nurses’ professional commitment to increase their intention to stay. The findings can serve as reference for developing newly graduated male nurse retention programs.

## 1. Introduction

The shortage of nursing staff is a critical global public health issue [1]. It must be highlighted and discussed urgently. It impacts the medical care system and increases the rates of mortality, infection, and medication error, and days of hospital stay of patients [2,3]. Although male nurses are still a minority in Taiwan, the proportion has been increasing continuously in recent years (from 0.8% in 2008 to 3.6% in 2021) [4]. However, this percentage is still far lower than those in European and American countries (e.g., 9.6% in the United States [5], 7% in Canada [6], and 10.7 in the United Kingdom [7]). In the foreseeable future, a high number of male nurses are expected to be engaged in nursing work in Taiwan. This will increase the diversity of nursing workplaces and alleviate the imbalanced male-to-female ratio in nursing workplaces [8].

Compared to female nurses, male nurses have a higher turnover rate and shorter retention time [9,10,11]. The turnover rate of male nurses is twice that of female nurses [9]. Most males leave the nursing profession within four years after starting their nursing career [10], while the average retention time of female nurses in the nursing profession is 10.29 years [11]. The transition process from school to nursing workplace is the most stressful period for newly graduated nurses; this phase witnesses the highest turnover rate [12]. It is important to help newly graduated male nurses adapt to the workplace and remain in the nursing profession at the early stage of their career.

No study has explored the influencing factors of newly graduated male nurses’ retention thus far. However, several studies have investigated the factors influencing nurses’ retention. Previous studies confirmed the predictor role of social support, resilience and nursing professionals’ commitment towards nurses’ intention to stay [13,14,15]. Social support and resilience can reduce job stress and turnover rate during career transition and increase professional commitment as well as psychological and social well-being [15]. Bernabe and Botia found that social support is an antecedent factor of resilience, and through social support, resilience and mental health (including stress and job burnout) are improved [16]. The factors affecting the retention of nurses are diverse and complex. The research gaps in the existing literature on the retention of nurses are as follows: (1) most studies ignored the complicated relationship as mediating or moderating effect among variables; (2) only a few theoretically based studies were investigated; and (3) most studies focused on all nurses and failed to consider the differences between genders, especially for male nurses. Male nurses (who are a minority in nursing) face more difficulties than female nurses when they enter the nursing profession [17]. In this study, the Job Demands-Resources model (JD-R model) was selected as the theoretical framework. The factors influencing the intention to stay of newly graduated male nurses was explored using a structural equation model (SEM).

The JD-R model has been widely used in different studies and different occupations to define specific factors related to employees’ work performance [18,19]. These specific factors include job demands and job resources. Job demands refer to things that must be done in work or work demands that need to continuously consume energy or resources, such as work stress and emotional exhaustion. It may affect the physiological and psychological state of employees and lead to health problems. Job resources refer to physical, psychological, social, and organizational incentives such as social support, organizational commitment, and job satisfaction. It can promote employees’ work motivation, work engagement, personal growth, and personal well-being. Recently, this model was modified by adding personal resources factors which are defined as positive self-evaluations that are linked to resilience and refer to individuals’ sense of ability to control and have an impact upon their environment successfully [18]. A systematic literature review confirms the effect of resilience on reducing job stress and burnout and indicates that personal resources mediated job demands, resources, and work engagement [2]. Overall, the JD-R model clearly explains the different interactions among job resources, personal resources, and job demands It indicated that work engagement or performance are governed by complex interactions [18]. The two research questions of this study were as follows: (1) What are the relationships among social support, resilience, nursing professional commitment and intention to stay of newly graduated male nurses? (2) Is the JD-R model applicable in explaining the relationships among social support, resilience, nursing professional commitment, and intention to stay?

## 2. Materials and Methods

### 2.1. Hypothetical Model

**Figure 1:**
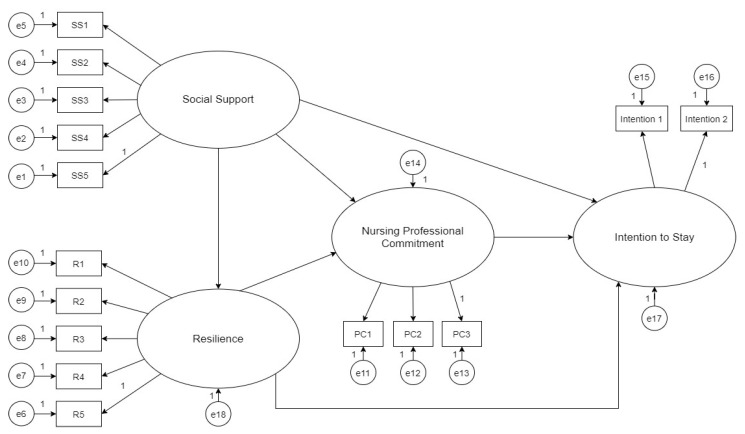
A structural equation model of the factors affecting the intention to stay in nursing of newly graduated male nurses.

Based on the JD-R model, this study tested the model on factors affecting the intention to stay in newly graduated male nurses in Taiwan. We proposed the structural equation model where the relationships among social support, resilience, nursing professional commitment, and intention to stay were explored. Furthermore, the mediating effect of nursing professional commitment in the above relationships was tested. This study assumes that social support and resilience of newly graduated male nurses positively influence nursing professionals’ commitment and intention to stay. Nursing professional commitment is a mediator between social support and intention to stay; it also acts as a mediator between resilience and intention to stay. Nursing professional commitment positively influences intention to stay. These relationships are graphically presented in Figure 1.

Nine hypotheses were formulated. These are as follows:

**Hypothesis** **1.** *The intention to stay model has an acceptable goodness of fit as tested by SEM.*

**Hypothesis** **2.** *Social support has a positive influence on resilience.*

**Hypothesis** **3.** *Social support has a positive influence on nursing professional commitment.*

**Hypothesis** **4.** *Social support has a positive influence on intention to stay.*

**Hypothesis** **5.** *Resilience has a positive influence on nursing professional commitment.*

**Hypothesis** **6.** *Resilience has a positive influence on intention to stay.*

**Hypothesis** **7.** *Nursing professional commitment has a positive influence on intention to stay.*

**Hypothesis** **8.** *Nursing professional commitment has a significant mediating effect on the relationship between social support and intention to stay.*

**Hypothesis** **9.** *Nursing professional commitment has a significant mediating effect on the relationship between resilience and intention to stay.*

### 2.2. Design, Setting, and Participants

This cross-sectional research used an online structured questionnaire to collect data. Newly graduated male nurses have a clinical seniority of two years or less after graduating [20]. Rigdon suggested that the sample size for structural equation models should be more than 150 [21]. Considering that the research response rate of male nurses was 83.2%, and the questionnaire completion rate was 92% in the past study [22], the estimated sample size in this study was at least 196. The inclusion criteria of newly graduated male nurses are as follows: (1) currently engaged in nursing work; (2) clinical seniority of two years or less; (3) conscious and able to understand and answer the researchers’ questions; (4) able to understand Chinese by listening or reading; and (5) willing to participate in this study and having signed the consent form. Exclusion criteria consisted of being unwilling to participate in this study. Since the newly graduated male nurses were distributed in 479 hospitals and other institutions in Taiwan (including clinics, health centres, and long-term care facilities), purposive and snowball sampling methods were used in this study. Male nurses were recruited from cooperative hospitals (two medical centres and two regional hospitals). Simultaneously, male nurses from other hospitals or other institutions were recruited by snowballing. In total, there were 272 valid surveys returned.

### 2.3. Measures

Five self-report structured questionnaires were used in this study.

#### 2.3.1. Participant Characteristics

The data of age, clinical seniority, education level, marital status, type of organisation, and working discipline were collected.

#### 2.3.2. Personal Resource Questionnaire (PRQ2000)

This 15-item questionnaire developed by Weiner [23] was used to measure the degree of social support perceived by newly graduated male nurses. The questionnaire has been translated into six languages and used in more than thirty-five countries [23]. It included five dimensions: attachment, reassurance of worth, social integration, nurturance, and availability, and assistance. A seven-point Likert-type scale (where 1 indicated ‘strongly disagree’ and 7 ‘strongly agree’) was designed for data collection. The total scores range from 15 to 105 with higher scores indicated the higher degree of social support. For validity and reliability of PRQ2000, the internal consistency Cronbach’α is 0.87–0.93, and the correlation of items is 0.38–0.70 [23]. Construct validity analysis shows that it is negatively correlated to the Center for Epidemiological Studies Depression (CESD) (r = −0.51, *p* < 0.001) [23]. The results of confirmatory factor analysis (CFA) in this study revealed the model χ^2^ = 2896, df = 5, *p* = 0.000, the goodness of fit index (GFI) = 0.957, the root mean square error of approximation (RMSEA) = 0.133, the comparative fit index (CFI) = 0.978.

#### 2.3.3. Connor-Davidson Resilience Scale (CD-RISC)

This 25-items scale, developed by Connor and Davidson [24], measured the resilience in the past month of newly graduated male nurses. It is the most used scale to measure resilience. A five-point Likert-type scale where 0 indicated ‘not true at all’ and 4 ‘true all the time’ was designed to collect the data. The total scores range from 0 to 100. The higher the score indicated the higher resilience. For validity and reliability, the internal consistency Cronbach’s α is 0.89 and the test-retest reliability is 0.87 in original scale [24]. Exploratory factor analysis (EFA) shows that all items can be divided into five dimensions [24]. The results of CFA in this study revealed the model χ^2^ = 1646, df = 5, *p* = 0.006, GFI = 0.977, RMSEA= 0.092, CFI = 0.988.

#### 2.3.4. Professional Commitment Scale

The scale developed by Meyer et al. [25] was used to measure professional commitment of newly graduated male nurses. The original scale was developed specially for nurses and has been used in several studies [26]. This eighteen-item scale included three dimensions: affective commitment (six items), continuance commitment (six items), and normative commitment (six items). A five-point Likert-type scale (where 1 indicated ‘strongly disagree’ and 5 ‘strongly agree’) was used to collect data. Higher scores indicated higher professional commitment [27]. For reliability and validity, the EFA show that it can be divided into three dimensions. The CFA revealed that RNI values are between 0.836 and 0.979, and PNFI values are between 0.738 and 0.845 in the original scale [25]. Discriminant analysis shows that it has discriminating power for organisational commitment [25]. The results of CFA in this study revealed the model χ^2^ = 2896, df = 5, *p* = 0.000, GFI = 0.957, RMSEA = 0.133, CFI = 0.978.

#### 2.3.5. Intention to Stay in Nursing

Intention to stay in nursing refers to the possibility that newly graduated male nurses will continue clinical nursing work in the future. It was measured by two questions (I want to remain in the nursing profession continuously in future for one year and three years) rated on a 10-point Likert scale (1 indicating ‘strongly disagree’ and 10 indicating ‘strongly agree’) [28].

### 2.4. Data Collection

First, the researchers explained the research purpose and methods to the newly graduated male nurses by means of posters and hospital social networking sites. After male nurses agreed to participate in this study, informed consent was sent to them, and they were requested to complete a web-link online questionnaire. It took about 30 min to fill out the questionnaire. For those who had not replied, they were urged for the first time by e-mail or telephone two weeks later. If they still had not replied, telephone contact was made two weeks later. If necessary, a paper questionnaire was sent, or a telephone interview was arranged to assist them in completing the questionnaire.

### 2.5. Ethical Consideration

This study was reviewed and approved by the Institutional Review Board (IRB No. 201901278B0). The purpose of the research was explained, and confidentiality was assured to uphold the rights pertaining to informed consent and confidentiality. All of the participants were informed that they had the right to withdraw from the study at any time. The questionnaires were anonymous, and no individual answers were discussed in this study. The participants were assured that on completion of the study, the written data and all identifiable personal information would be destroyed, and the relevant research data would be stored for 10 years.

### 2.6. Statistical Analysis

Statistical analyses were performed using IBM SPSS Statistics (Statistical Package for the Social Science) for Windows Version 26.0 and AMOS 18.0. The SEM methodology was used to test the propose model using the model fit criteria proposed by Bagozzi and Yi, which include preliminary model fit criteria, overall model fit, and fit of internal structure of model [29]. In addition, CFA was used to evaluate the factor structures for the instrument.

## 3. Results

This section may be divided by subheadings. It provides a concise and precise description of the experimental results, their interpretation, and the experimental conclusions.

### 3.1. Participant Characteristics

Participant characteristics for the 272 newly graduated male nurses in this study are shown in Table 1. The mean age was 27.54 years (Mean ± SD = 27.54 ± 6.50), and the average clinical seniority was 14.61 months (Mean ± SD = 14.61 ± 6.50). In terms of education level, most of them graduated from universities (68.4%), followed by junior college (30.1%). About 82.0% of participants were single. Those working in regional hospitals accounted for the highest proportion (39.3%), followed by medical centres (37.9%). In terms of the working discipline, intensive care unit (ICU) accounted for the highest proportion (21.0%), followed by emergency department (19.1%), and psychiatric wards (11.4%).

### 3.2. Social Support, Resilience, Nursing Professional Commitment, and Intention to Stay of Newly Graduated Male Nurses

The newly graduated male nurses had a high level of intention to stay (Mean ± SD = 7.78 ± 2.08, range = 1–10). The intention to stay for one year was 8.04 (SD = 2.09), and the intention to stay for three years was 7.52 (SD = 2.21). They displayed a high level of social support (Mean ± SD = 5.99 ± 0.69, range = 1–7), a moderate level of resilience (Mean ± SD = 3.93 ± 0.54, range = 1–5), and a moderate level of nursing professional commitment (Mean ± SD = 3.55 ± 0.51, range = 1–5).

### 3.3. Test of the Research Model

This study evaluated the SEM of the factors influencing newly graduated male nurses’ intention to stay. For the goodness of fit evaluation criteria of the model, the preliminary model fit criteria, overall model fit, and fit of internal structure of model proposed by Bagozzi and Yi [29] were adopted. Moreover, we also referred to the cut-off values of goodness of fit index proposed by Hair et al. [30] since their guidelines have stricter standards for acceptable fit. The following, respectively, describes the model fit evaluation results.

#### 3.3.1. Preliminary Model Fit Criteria

The ‘preliminary fit criteria of model’ proposed by Bagozzi and Yi was used as the measurement model fit test in this study [29]. According to the parameter estimation of the theoretical model of intention to stay, none of the standard error variance estimates was negative, and the standard error variance ranged from 0.47 to 0.99 (*p* < 0.01). In addition, the factor loadings of each observed variable ranged from 0.55 to 0.94 (*p* < 0.01), which was within the range of from 0.5 to 0.95 as recommended by Bagozzi and Yi. Therefore, the theoretical framework of intention to stay proposed in this study agrees with the preliminary fit criteria of model (Table 2).

#### 3.3.2. Overall Model Fit

Overall model fit was used to evaluate the fit between the research data and theoretical framework. Table 3 shows that the chi-square of the overall model fit between the theoretical model and the data was 242.61 (df = 86), *n* = 272, *p* = 0.000, which is statistically significant. The goodness of fit index (GFI), adjusted goodness-of-fit index (AGFI), normal fit index (NFI), incremental fit index (IFI), and non-normed fit index (NNFI) were also included. The result showed that the GFI was 0.90, the AGFI was 0.85, the NFI was 0.92, the IFI was 0.95, and the NNFI was 0.94. All of these values (except for AGFI) were over 0.90, indicating a better fit. Thus, the model in this study can explain the newly graduated male nurses’ intention to stay in nursing. As for residual analysis, the RMSEA is the residual index of the overall model, and in this model, its value is 0.08, which reaches the evaluation standard. Therefore, the fit of the model is acceptable. In general, the fit indices demonstrated an ideal external quality.

#### 3.3.3. Fit of the Internal Structure of the Model

The fit of internal structure of model can be used to evaluate whether the internal quality of the measurement model and structural model meets specific standards [29]. Regarding the evaluation of measurement mode, Hair et al. suggested that factor loadings of observation variables greater than 0.70 are better for individually observed index reliabilities below 0.5. In this study, only four individual indexes out of the fourteen observation variables of the theoretical model had an individual reliability much below 0.50. Furthermore, the reliabilities of the remaining eleven observation variables were between 0.62 and 1.00.

In addition, Fornell suggested that the composite reliability of potential variables must be greater than 0.6 [31]. In this study, the composite reliability of four latent variables (social support, resilience, nursing professional commitment, and intention to stay) were 0.93, 0.91, 0.67, and 0.93, respectively, all greater than 0.6. According to Fornell and Larker, the average variance extracted (AVE) of potential variables must be greater than 0.5 [32]. The results showed that only the AVE value of nursing professional commitment was 0.41, while the AVE values of other latent variables were greater than 0.5. Overall, the various model-fit criteria in this study were still above the standard; therefore, hypothesis H1 was supported.

#### 3.3.4. Effects on the Latent Variables of Intention to Stay in the Theoretical Model

**Figure 2:**
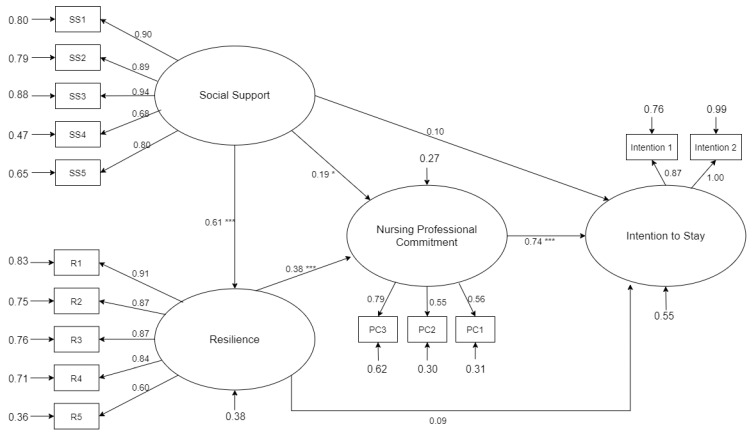
Effects on the latent variables of intention to stay in the theoretical model. * *p* < 0.05, *** *p* < 0.001.

As shown in Figure 2, the direct correlations of social support with resilience, nursing professional commitment, and intention to stay were 0.61 (*p* < 0.001), 0.19 (*p* < 0.05), and 0.10 (*p* > 0.05), respectively. Only the last correlation did not reach a significance level of 0.05. Therefore, hypotheses H2 and H3 was supported, while hypothesis H4 was not. Furthermore, the direct effects of resilience on nursing professional commitment and intention to stay were 0.38 (*p* < 0.001) and 0.09 (*p* > 0.05), respectively. Only the former reached the 0.05 significance level. Thus, hypothesis H5 was supported, and H6 was not. Finally, the direct effects of nursing professional commitment on intention to stay were confirmed and the correlation was 0.74 (*p* < 0.001), thereby supporting hypothesis H7.

Furthermore, based on the result, nursing professional commitment could serve as a complete mediator between the relationship between social support and intention to stay and the relationship between resilience and intention to stay. Hence, Hypothesis H8 and H9 were supported.

## 4. Discussion

This study had several contributions. First, it compensated for the insufficient literature on the factors influencing the intention to stay of newly graduated male nurses. It found that the intention to stay of newly graduated male nurses was high, regardless of the intention to stay for one year or three years. Second, with respect to the influencing factors on intention to stay (based on the JD-R model), the obtained structural equation model revealed a path model. This allowed us to examine and clarify the correlations between various variables and intention to stay. It also confirmed the complete mediating effect of nursing professional commitment on the relationship between social support and intention to stay and between resilience and intention to stay. Third, the results of this study showed that the preliminary model fit criteria, overall model fit and fit of internal structure of model were acceptable. Therefore, the theoretical model of intention to stay of male nurses and the observed values of model fit criteria were acceptable. The applicability of the JD-R model in the newly graduated male nurse was verified in this study.

This study found that the intention to stay of newly graduated male nurses was high, regardless of the intention to stay for one year or three years. The results of this study were the same as the findings of Lou et al. [33]. The study by Lou et al. showed that male nurses with zero to two years of clinical seniority had the lowest turnover intention, while the nurses with four to five years of clinical seniority had the highest turnover intention [33]. The reasons for the high intention to stay of newly graduated male nurses in Taiwan may be related to the increasing salary of nurses and the reduction of gender role stereotypes among the public, and male nurses actively facing up to dilemmas and receiving positive support from family members, friends, female nurses, and supervisors [34,35]. Moreover, the implementation of retention strategies for newly enrolled nurses in recent years (such as the Preceptor Program Model, New Graduated Nurse Focus, Post Graduate Year program (PGY), and the externship program) was also helpful [36]. There have been many interventions to assist newly graduated nurses remaining in the nursing profession. However, Labrague and McEnroe-Petitte indicated that the intervention for newly graduated nurses during the transition period were still insufficient, and that most intervention studies lacked structural and complete design [37]. In addition, the current retention intervention for new nurses mainly considers improving clinical ability as well as linking training between school and clinical practice. Very few studies focus on the difficulties faced by male nurses, which are discussed hereafter. First, due to traditional gender stereotypes, they are usually considered feminine [38]. Second, Tzeng and Chen [39] suggests that males cannot fully dedicate themselves to basic nursing work; once their employment environment stabilizes, they are likely to start thinking about their future. However, most male nurses lack career advice from nursing professionals or guidance counsellors [35], resulting in the lack of planning for their own nursing career. Third, they are present in a female-dominated nursing care scenario and lack male role models [40]. Fourth, in the traditional Taiwanese culture, the idea of men taking care of young women still cannot be easily accepted by patients and their families [34]. Therefore, male nurses face the dilemma of their care being refused or being unwelcome among patients [41]. Fifth, they are mistaken as doctors [35]. Sixth, at the early stage of their career, they can easily become the subjects of attention in the female-dominated nursing workplace; this may lead to higher professional expectations, thereby causing stress [35]. Therefore, it is necessary to carry out more intervention studies on retention strategies for newly graduated male nurses.

The research results of the SEM in this study confirmed that nursing professional commitment had a highly positive influence on the intention to stay. It played a complete mediating role between social support and intention to stay, as well as between resilience and intention to stay. This result emphasizes the mutual influence between male nurses’ nursing professional commitment and their intention to stay. Several extant studies on different occupations have confirmed the significant correlation between organizational or professional commitment and retention [34,42,43,44,45]. However, the causal relationship is uncertain. Therefore, further research is required to confirm the causal relationship. In addition, Garcı’a-Moyano et al. believed that in order to promote the formation of nursing professional commitment, besides material resources (such as rewards and salary) we need to increase more personal motivation for professional development and cultivate actual professionals in nursing [46]. Cheng et al. pointed out that newly graduated male nurses are often concerned about their lack of ability and doubt whether they are suitable for nursing. In addition, based on the expectation of traditional gender roles for males, most male nurses are highly concerned about their career choice and development as well as the possibility of promotion [34]. Therefore, allowing newly graduated male nurses to reflect their nursing professional ability and the significance of their nursing, and encouraging them to develop various possibilities of nursing career, such as nursing management and becoming a nurse practitioner or clinical nurse specialist, may contribute to the formation of nursing professional commitment among newly graduated male nurses.

The results of this study revealed that there was a positive correlation between social support and resilience of newly graduated nurses, which were the same as the results of Öksüz et al. [47] and Wang et al. [48]. Several theories have highlighted the mechanism by which social support promotes resilience. For example, Ozbay et al. believed that the connection between social support and resilience involves many neurocognitive systems and genetic mechanisms [41]. Previous neurocognitive and genetic research has found that the existence of companions can inhibit behavioural and physiological stress responses, whether in humans or animals. At this time, the parasympathetic nervous system and brain areas are activated, and the response of stressors decrease. At the same time, the secretion of neuropeptide and oxytocin increased, and the activities of SNS and HPA-axis decrease [49]. In addition, according to the attachment theory, attachment can directly and indirectly regulate self-awakening and stress response. Higher social support can increase the sense of belonging and solidarity as well as encourage healthy coping behaviour. It can help individuals redefine difficult situations and enhance the regulation of distrust, anxiety, and fear [50]. According to the resilience model theory, the development of psychological resilience contains two essential elements, the internal protection factor and external protection factor [51]. Therefore, social support as an external protection factor will promote the development of resilience. Previous research has found that newly graduated male nurses have insufficient social support in nursing practice choice, career development, career promotion [13,52], and dealing with embarrassing clinical situations caused by gender (such as close physical contact with patients) [53]. Therefore, providing social support such as career development and promotion suggestions, as well as mentors or peers for newly graduated male nurses, may be benefited to enhance individuals’ resilience.

This study showed that newly graduated male nurses perceive a high degree of social support and a moderate degree of resilience, and both social support and resilience are positively correlated with nursing professional commitment. In Taiwan, nursing traditionally has been viewed as a woman’s profession. The nursing discipline was not male nurses’ best career preference [13]. The positive aspects of nursing (included job stability, an acceptable salary, and a profession that helps persons) and academic achievement and/or the college entrance exam contributed to the reason that the men chose to become a nurse [35]. A previous study indicated that male nurse perceived job stressors not only in work overload and resource inadequacy, but also included role conflict and role ambiguity [54]. Social support was the major resource that nurses identified that they needed when dealing with job stressors. When male nurse got social support from a supervisor, a co-worker, or family members, their job stressors decreased and nursing career developed [13,54]. It may help them to adapt to their professional settings better and develop their nursing professional commitment. In addition, because of society’s expectation of gender roles, males are expected to have individuality, strength, independence, and invincibility [55]. Male nurses are more likely to adopt positive coping strategies compared to female nurses when encountered with adversity [56]. Therefore, compared with females, males scored higher in resilience [57]. However, in this study, the resilience of male nurses was moderate. This might have been affected by the unfriendly nursing environment, heavy workload, unsatisfied pay, and limited opportunities for promotion, which will wear out their resilience and affect their professional commitment. In the future, we suggest that there should be more gender studies on nursing professional commitment.

### Limitation

This study had several limitations. First, it verified the applicability of the JD-R model in newly graduated male nurses. Since the research subjects were newly graduated male nurses in Taiwan, the application in different regions and cultures requires further verification. Second, this is a cross-sectional study and SEM methodology was adopted to test propose model. More longitudinal design research or intervention research are necessary in the future for drawing the causal relationship that this study could not. Third, most newly graduated male nurses are young and extensively use social networking sites. An online questionnaire was used to collect data. Although this process could increase response rate and minimize missing data, individual queries of participants could not be answered in time. Moreover, we could not prevent and identify some unwanted persons who were interested in this questionnaire completing it. Finally, the sampling bias may cause by purposive and snowball sampling in this study. The applicability to other settings is limited. Random sampling method is suggested to future study.

## 5. Conclusions

In this study, an SEM clarified the relationships among social support, resilience, nursing professional commitment, and intention to stay. The applicability of the JD-R model in the intention to stay of newly graduated male nurses was verified. In addition, this study confirmed the complete mediating effect of nursing professional commitment between social support and intention to stay, as well as between resilience and intention to stay. Nursing professional commitment had a high positive correlation with the intention to stay. It is suggested that future research and practice should enhance male nurses’ professional commitment to increase their intention to stay. Besides the provision of material resources, such as salary and reduced workload, increasing male nurses’ professional development motivation and encouraging them to develop multiple possibilities for their nursing career may be helpful.

## Figures and Tables

**Table 1:** Participant characteristics of newly graduated male nurses (*n* = 272).

Variables	*n*	%
Education level		
Junior college	82	30.1
Undergraduate	186	68.4
Graduate school	4	1.5
Marital status		
Single (including widowed, unmarried, divorced, etc.)	223	82.0
Married	49	18.0
Type of organisation		
Medical centre	103	37.9
Regional hospital ^a^	107	39.3
District hospital ^b^	30	11.0
Other	32	11.8
Working discipline		
Operating room	26	9.6
Emergency department	52	19.1
Intensive care unit(ICU)	57	21.0
Medical wards	28	10.3
Surgical wards	18	6.6
Psychiatric wards	31	11.4
Paediatric wards	16	5.9
Outpatient	16	5.9
Other ^c^	28	10.2

Notes: a: Regional hospital = acute general beds + acute mental beds = 100–249, and total bed number >300; b: District hospital = acute general beds + acute mental beds <99 beds; c: Other = clinic, nursing home, public health centre, long term care, hospice.

**Table 2:** Analysis results of preliminary fit in the theoretical model of intention to stay (*n* = 272).

Evaluation Items	Analyses Results	Evaluation Results
Were there any negative error variances?	Standardized error variances were between 0.47 and 0.99.	Yes
Did error variances reach significance levels?	Reached the 0.01 significance level.	Yes
Were the related absolute values among the parameters close to 1?	Maximum absolute value among the parameters was 1.0.	Yes
Were factor loadings between 0.50 and 0.95?	Factor loadings of observation variables were between 0.55 and 0.94.	Yes

**Table 3:** Model evaluation measures of the overall model fit (*n* = 272).

Evaluation Items	Analyses Results	Evaluation Results
Did χ^2^ reach significance (*p* < 0.05)?	χ^2^ = 242.61, *p* = 0.000	Yes
Did χ^2^/*df* < 3?	2.8	Yes
Did GFI > 0.90?	0.90	Yes
Did AGFI > 0.90?	0.85	Not satisfactory
Did RMR < 0.05	0.067	Not satisfactory
Did Δ_1_ (NFI) > 0.90?	0.92	Yes
Did Δ_2_ (IFI) > 0.90?	0.95	Yes
Did TLI (NNFI) > 0.90?	0.94	Yes
Did RMSEA < 0.08?	0.08	Yes
Did CFI > 0.90?	0.95	Yes

## Data Availability

The data presented in this study are available on request due to privacy restrictions.
